# A Global Assessment of Salmon Aquaculture Impacts on Wild Salmonids 

**DOI:** 10.1371/journal.pbio.0060033

**Published:** 2008-02-12

**Authors:** Jennifer S Ford, Ransom A Myers†

**Affiliations:** Department of Biology, Dalhousie University, Halifax, Nova Scotia, Canada; University of York, United Kingdom

## Abstract

Since the late 1980s, wild salmon catch and abundance have declined dramatically in the North Atlantic and in much of the northeastern Pacific south of Alaska. In these areas, there has been a concomitant increase in the production of farmed salmon. Previous studies have shown negative impacts on wild salmonids, but these results have been difficult to translate into predictions of change in wild population survival and abundance. We compared marine survival of salmonids in areas with salmon farming to adjacent areas without farms in Scotland, Ireland, Atlantic Canada, and Pacific Canada to estimate changes in marine survival concurrent with the growth of salmon aquaculture. Through a meta-analysis of existing data, we show a reduction in survival or abundance of Atlantic salmon; sea trout; and pink, chum, and coho salmon in association with increased production of farmed salmon. In many cases, these reductions in survival or abundance are greater than 50%. Meta-analytic estimates of the mean effect are significant and negative, suggesting that salmon farming has reduced survival of wild salmon and trout in many populations and countries.

## Author Summary

The impact of salmon farming on wild salmon and trout is a hotly debated issue in all countries where salmon farms and wild salmon coexist. Studies have clearly shown that escaped farm salmon breed with wild populations to the detriment of the wild stocks, and that diseases and parasites are passed from farm to wild salmon. An understanding of the importance of these impacts at the population level, however, has been lacking. In this study, we used existing data on salmon populations to compare survival of salmon and trout that swim past salmon farms early in their life cycle with the survival of nearby populations that are not exposed to salmon farms. We have detected a significant decline in survival of populations that are exposed to salmon farms, correlated with the increase in farmed salmon production in five regions. Combining the regional estimates statistically, we find a reduction in survival or abundance of wild populations of more than 50% per generation on average, associated with salmon farming. Many of the salmon populations we investigated are at dramatically reduced abundance, and reducing threats to them is necessary for their survival. Reducing impacts of salmon farming on wild salmon should be a high priority.

## Abstract 3

Comparing the survival of wild salmonid populations in areas near salmon farms with unexposed populations reveals a large reduction in survival in the populations reared near salmon farms.

## Introduction

Since the late 1970s, salmon aquaculture has grown into a global industry, producing over 1 million tonnes of salmon per year [1]. The majority of this biomass is held in open net pens in coastal areas through which wild salmon migrate on their way to and from the ocean. A number of studies have predicted or evaluated the impacts of salmon farming on wild salmon through a single mechanism, in a given area. It is clear that some salmonids are infected and killed by sea lice originating from salmon farms [2–5], that other diseases have been spread to wild populations from salmonid farming activities [6,7], and there is evidence that salmon parr are at lower density in areas of Scotland where there is salmon aquaculture [8]. In addition, farmed salmon escape in all areas where salmon aquaculture is practiced, and although their breeding success may be low on average, competition for mates and hybridization with wild salmon are likely to reduce survival of wild populations [9,10].

**Figure 1:**
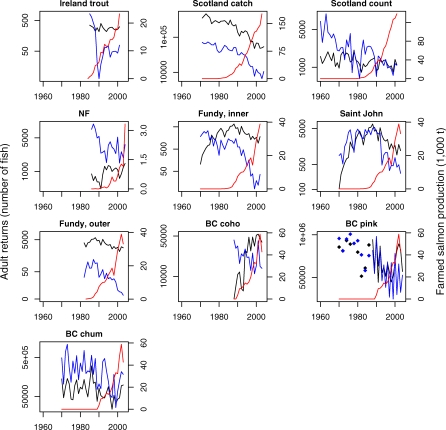
Adult Returns of Wild Salmonids in Control (Black) and Exposed (Blue) Stocks, with Aquaculture Production (Red) For plotting only, the returns to controls and exposed stocks have been separately summarized by a multiplicative model (log(*Returns_i_*_,*y*_) = *a_i_* + *d_y_* + *e_i_*_,*y*_ ; variables are the same as in Equation 1). The mean returns across stocks for each year are shown. Note that left-hand axes are on a log scale. Only even year values are available for pink salmon prior to 1989. Irish salmon are not included because only marine survivals (not returns) are available.

It is well established that wild salmonids can be negatively affected by salmon farming [11], however, the importance of these interactions at the population level has rarely been determined [2]. To determine population level impacts, we examined temporal trends in the abundance and survival of wild salmonids (Figure 1 and Figure S1). Our study contrasted trends in wild populations exposed to potential aquaculture impacts with those of populations not exposed. Populations in which juvenile salmonids pass by salmon farms during their migration were considered to be exposed to impacts of salmon farming. Exposed populations were carefully paired with control populations in the same region whose migrations did not lead past farms, but which otherwise experienced similar climate and anthropogenic disturbances. Use of such paired comparisons allowed us to control for confounding factors such as climate to detect population level impacts. Using the Ricker stock recruit model [12], we performed 11 comparisons, involving many stocks from both sides of the Atlantic and from British Columbia in the Pacific (Table 1, Data section of Materials and Methods).

**Table 1:**
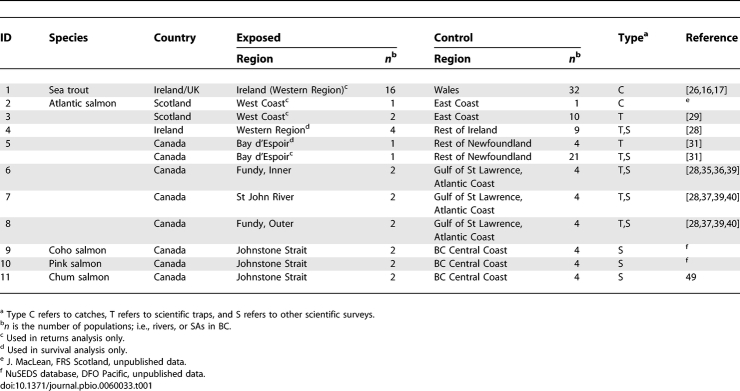
Summary of Populations Included

## Results

**Figure 2:**
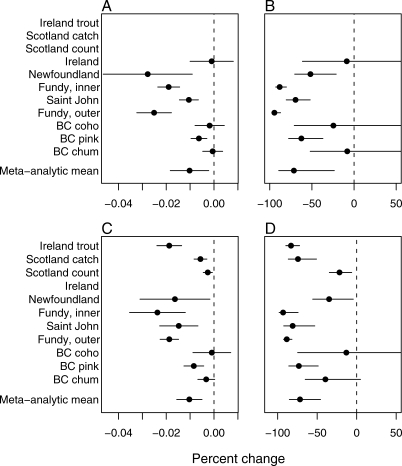
Estimated Effects of Salmon Farming All estimates are for Atlantic salmon unless otherwise noted. (A) Estimated percent change in survival of wild salmonids associated with salmon farming, per generation per tonne of farmed salmon production. (B) Estimated percent change in survival of wild salmonids associated with salmon farming, per generation, at the mean tonnage of farmed salmon harvested in each region, during the study period. The meta-analytic mean has been scaled to show mean reduction in survival when harvest of farmed salmon in the region is 15,000 t. (C and D) As for (A) and (B), but representing the change in returns to each stock (rather than survival). The bars represent 95% confidence intervals.

All estimates of the effect of aquaculture on survival or returns were negative. Both random effects estimates of the mean effect were negative and highly significant (Figure 2), indicating a very large reduction in survival and returns in populations exposed to aquaculture. Under the dynamics of Equation 1 (see Materials and Methods), percent change in survival or returns is represented by 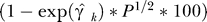 where γ is the coefficient of aquaculture production (*P*) for region *k*. For example, the estimated change in survival per tonne of salmon farming (γ*_k_*) for Bay d'Espoir in Newfoundland was estimated to be 0.026 (Figure 2). In 2003, the farmed salmon harvest from this area was 1,450 tonnes (t), so the estimated decrease in survival is 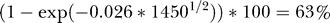 (95% CI: 44%–80%), relative to what it would be in the absence of farms. Survival and total returns of many stocks were found to be reduced by more than 50% (Figure 2), for each generation. If all exposed populations were passing by farms with a total annual harvest of 15,000 t, the mean estimated total reduction in survival would be 73% (95% CI: 29%–90%) (Figure 2). Many regions now have farmed salmon production in excess of 20,000 t/y.

Generally, Atlantic salmon populations were depressed more than Pacific salmon populations, particularly Atlantic salmon in Atlantic Canada. Irish sea trout were also estimated to have been very strongly reduced by impacts of salmon farming, whereas estimated impacts on Atlantic salmon in Scotland depended on the data used. In British Columbia (Pacific Canada), only pink salmon showed significant declines correlated with salmon aquaculture.

Results are reported for a model including autocorrelated errors and with λ set at 0.5, rather than 1 or 2, because this minimized the Akaike information criteria (AIC) for most regions [13]. The parameter λ allows for the impacts of salmon farming to change nonlinearly with the aquaculture production. A λ of 0.5 indicates that relatively small amounts of aquaculture will depress wild populations, but the effect does not increase proportionally to aquaculture production. See Tables S1 and S2 for results of alternative models.

For the New Brunswick comparison, the outer Bay of Fundy rivers are located much closer to salmon farms than the other exposed rivers. If only these outer Bay of Fundy rivers are considered exposed to salmon farming, and other Bay of Fundy rivers (inner Bay of Fundy and Saint John River) are included among the controls, the overall estimates (i.e., meta-analytic means) are still significant and negative in both versions of the analysis.

## Discussion

We have estimated a significant increase in mortality of wild salmonids exposed to salmon farming across many regions. However, estimates for individual regions are dependent on assumptions detailed in the Materials and Methods section, and the estimates often have large confidence intervals. Given that the data analysed are affected by considerable noise—including changes in fishing and environmental factors—the important result of this study is that we are nonetheless able to detect a large, statistically significant effect correlated with trends in farmed salmon production. The significant increase in mortality related to salmon farming that we have estimated in almost all cases is in addition to mortality that is also acting on the control populations. In most cases, control populations were also experiencing decreases in marine (and sometimes freshwater) survival, for reasons that are only partially understood. At the same time, fishing mortality has been reduced or eliminated in many areas, which may have partially masked high mortalities associated with aquaculture.

A key assumption in this study is that exposed and control areas do not differ in a systematic way across regions. We have identified three possible ways that exposed and control sites could differ systematically: first, salmon farms could be established only in areas where wild stocks have already collapsed; second, salmon farms could be established in areas where habitat is more disturbed by human activities; or, third, climate factors could differ between the exposed areas and the controls in a systematic way.

Declines in control and exposed salmonid populations preceded the growth of the salmon aquaculture industry in some regions, but inspection of the data used do not indicate that salmon populations in the majority of our regions had declined dramatically in the exposed areas only, before the start of salmon farming (averaged returns data are shown in Figure 1). In regions such as Scotland, where declines precede the start of salmon farming, the strong aquaculture effect estimated reflects a faster decline in exposed populations concurrent with the growth of salmon farming.

Areas that we consider exposed do not seem to be more developed than control areas in general. In the Atlantic, most areas have been highly altered by human activities for hundreds of years, but there is no obvious difference between the control and exposed groups in this regard. In British Columbia, all areas considered are very remote, and the main type of anthropogenic disturbance in rivers would be forestry. Comprehensive forestry records at the watershed scale are not easily available, but logging in British Columbia's Central Coast is extensive, both historically and recently [14]. It should be noted that the comparisons in British Columbia include large numbers of rivers (> 80 rivers in each case), so differences in anthropogenic effects would have to hold over many watersheds to explain the effects we estimate.

Finally, it is also very unlikely that our results are due to a climate driven trend in which more southerly populations show stronger declines than populations to the north. Although our exposed populations are to the south of control populations in three of five regions, differences in latitude are small. In New Brunswick, the control populations are to the north of the exposed populations, but by less than 200 km, and the headwaters of some of the exposed populations are adjacent to those of the controls. In Newfoundland, the difference in latitude between exposed and control populations is similarly small. In British Columbia, the control populations are also to the north, but by less than 300 km. Also, Mueter et al. [15] found that pink and coho salmon from all of the British Columbia populations we have examined respond similarly to large-scale climate trends. Thus, the pattern we found in this study does not seem attributable to a systemic difference between the control and exposed areas.

We estimated higher impacts on populations in the Atlantic than those in British Columbia, possibly because Atlantic salmon populations are conspecific with farmed salmon, and therefore susceptible to genetic effects from interbreeding with escaped farm salmon, in addition to disease or other impacts. Estimated impacts in British Columbia may also be lower because we aggregated over large numbers of populations for pink, chum, and coho salmon, because estimates of fishing mortality were only available at a very coarse scale. The individual populations may vary in their exposure to salmon farms.

The large apparent impact of Atlantic salmon farming on Irish sea trout, in contrast, can not be explained by interbreeding. In the mid-western region of Ireland (the exposed region), the total rod catch decreased from almost 19,000 sea trout in 1985 to 461 in 1990 [16]. In the few rivers where data were available, catch declines could not be explained by reduced effort [16]. Welsh sea trout catches (the controls) have remained relatively constant during the same time period, whereas fishing effort has decreased considerably [17]. Sea trout (anadromous brown trout) might be expected to experience higher mortalities, because they spend lengthy periods in coastal areas near salmon farms, relative to Atlantic salmon, thus being exposed to disease or parasites for a longer time [18].

The time period over which we are estimating impacts of aquaculture includes the establishment of the industry in each region. Improvements in management as industries mature may explain our finding that impacts of salmon farming on wild salmon do not increase linearly with the tonnage of farmed salmon. Better management should decrease the impact of salmon farming on a per tonne basis, although such improvements may not be able to keep pace with the growth of the salmon farming industry. The estimated reduction in survival of wild salmonids is large, and would be expected to increase if aquaculture production increases.

## Materials and Methods

We modeled survival and, in a separate analysis, total returns to each stock, using a general linear mixed effects model for each region. To model survival, we used a Ricker model extended to include the production of farmed salmon in the area through which exposed juvenile salmon migrated, with random effects for each stock and year [19].

Let *S_i_*_,*y*_ be an index of the number of fish that smolted, i.e., migrated to sea in the spring, in year *y* from stock *i*, let *R_i_*_,*y*_ be the estimated number of those fish that would subsequently return to spawn in the absence of fishing, and let *P_i_*_,*y*_ be the aquaculture production that those smolts were exposed to (in tonnes). The dynamics are assumed to be given by

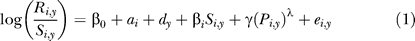
where β_0_ is the fixed intercept for the average stock and year with no aquaculture production, *a_i_* is the random deviation of the *i* ^th^ stock intercept from β_0_, *d_y_* is the random deviation of the *y* ^th^ year, β*_i_* is the fixed slope of mortality (the density dependence parameter) that will vary with each stock *i*, and γ is the coefficient of aquaculture mortality that is assumed to scale with a possibly nonlinear function of aquaculture production, (*P_i_*_,*y*_)^λ^. The random error, *e_i_*_,*y*_, is assumed to be first order autocorrelated. We assume the *a_i_*'s and *d_y_*'s come from normal distributions with zero mean. The autocorrelation and the random year effect are included to account for established temporal and spatial correlations (respectively) in environmental effects [20].

The effects of aquaculture are summarized by the coefficient γ for each region. The regional coefficients were combined using meta-analysis to obtain an overall estimate of the change in wild salmonid survival related to aquaculture. Because the best functional form for the aquaculture term in the model (*P_i_*_,*y*_)*^λ^* was not known, we investigated a linear increase in impacts with aquaculture, a square relationship, and a square root relationship. We selected models by AIC, and we tested our results under alternative formulations.

To test the robustness of the conclusions, and because only returns data were available for some regions, we repeated the analysis with number of returning adults as the response variable. This analysis used Equation 1 but dropped the *S_i_*_,*y*_ and β*_i_* terms. The response variables for this analysis included rod catches, rod plus marine catches, counts of salmon returning to rivers, and estimates of returns to rivers in the absence of fishing (see Data sources and treatment, below).

Outer Bay of Fundy salmon in New Brunswick, Canada, have been reduced to zero in one river and to a handful in another river. For this region only, we assumed negative binomial errors.

For the meta-analysis, we added a subscript, *k*, to identify each region, to γ, which summarizes the effect of aquaculture for each region. For a fixed assumption about λ, the γ*_k_*'s are in the same units and can be directly compared. We modeled the effects of aquaculture as a mixed effects model,

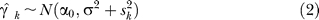
here 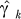 is the estimated value of γ*_k_, α*_0_ is the intercept, σ^2^ is the among-region variance, and 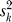 is the variance of the *k*th estimate (which is taken from the analysis in Equation 1, and is held fixed). A fixed effects meta-analysis is obtained by constraining σ to be zero. We used maximum likelihood estimation and selected models by AIC.

For robustness, we considered five classes of models: different regions used as controls, different mixed model assumptions, different error assumptions, different functional forms for the aquaculture effect, and different autocorrelational structures, as well as performing a Bayesian meta-analysis. Overall, the results were very similar for all models. (See Tables S1 and S2 for results of alternative models and Text S1 for details of the Bayesian analysis.)

### Data sources and treatment.

We analysed data for five species of wild salmonid in five regions: Ireland and Wales, Scotland, Newfoundland (Canada), New Brunswick (Canada), and British Columbia (Canada). There are three further regions with both wild salmonids and salmon aquaculture for which we could not carry out analyses: Norway, the west coast of Vancouver Island (Canada), and Maine (United States). We were unable to carry out analyses for Norway for three reasons. First, salmon farming in Norway is so widespread [21] that it was difficult to establish controls. Second, the adult population in many rivers has been found to contain over 50% aquaculture escapees [22], making trends in returns to rivers difficult to interpret. Third, there are confounding effects from acidification and disease [23, 24]. For the west coast of Vancouver Island, it was not possible to obtain aquaculture production data by region over time, and Maine was not included because of a lack of nearby wild populations to serve as controls.

Most populations that we considered to be exposed breed in rivers that discharge into bays or channels containing at least one salmon farm. Others breed in rivers flowing into bays without salmon farms very close to areas containing many farms. Salmon from control rivers are very unlikely to pass by salmon farms early in their life cycle, due to the direction of their migration. However, some controls may be relative, in the sense that salmon may pass by farms from a considerable distance, later during their migrations. This would tend to be conservative with respect to our study, since we would then have to detect local effects that are additional to any impacts from distant farms. Data from scientific surveys, e.g., counting fences, were used if possible; for Scottish salmon and Irish and Welsh sea trout, only catch data were available, so results are given for only the impacts on returns (not survival).

### Ireland sea trout.

We compared rod catches of sea trout in Ireland's Western Region to rod plus in-river fixed engine catches in Wales, from 1985 to 2001 (there are no fixed engine fisheries directed at sea trout in Ireland). Salmon farming is concentrated in the Western Region (Connemara area) of Ireland, but does occur in other parts of the country [25]. Based on farm locations [25], it was estimated that all rivers considered exposed are located less than 50 km from a salmon farm, but most will enter the ocean less than 30 km from a salmon farm. There is no salmon farming in Wales. There were 16 rivers in Western Ireland considered exposed: Athry, Bhinch (Lower), Bhinch (Middle), Bhinch (Upper), Burrishoole, Costello, Crumlin, Delphi, Erriff, Gowla, Inagh, Inverbeg, Invermore, Kylemore, Newport, and Screebe [16]. The following 32 Welsh rivers served as controls: Aeron, Afan, Arto, Cleddau, Clwyd, Conwy, Dee, Dwyfawr, Dwyryd, Dyfi, Dysynni, Glaslyn, Gwendreath, Gwyrfai, Llyfni, Lougher, Mawddach, Neath, Nevern, Ogmore, Ogwen, Rheidol, Rhymney, Seiont, Taf, Taff, Tawe, Teifi, Tywi, Usk, Wye, and Ystwyth [26,27]. Trout caught and released are included in catch data from both countries. Only catch estimates were available for most of these rivers. Recruitment could not be derived, because anadromous brown trout interbreed with freshwater resident trout, about which very few data are available, so this stock was only included in the returns modeling (not survival). Farmed salmon production for all of Ireland was used in modeling [28], because the majority of farms are in the region where the exposed populations breed. This will tend to have a conservative effect, resulting in a lower estimate of the impact of aquaculture, per tonne of salmon farming.

### Scotland catch data.

We compared marine plus rod catches of Atlantic salmon from the east coast of Scotland to catches from the west coast of Scotland for the years 1971 to 2004. Salmon farms appear to be located in the majority of bays on the west coast of Scotland in well over 300 sites (http://www.marlab.ac.uk/Uploads/Documents/fishprodv9.pdf), so all salmon from rivers on this coast were considered exposed. There is no salmon farming on the east coast, so salmon from east coast rivers were controls. For each coast, a single time series of total catch was used in modeling. Marine catch records were from the International Council for the Exploration of the Sea (ICES) Working Group on North Atlantic Salmon [28] and rod catch records were from Fisheries Research Services of Scotland (J. MacLean, personal communication). Rod catches included salmon caught and released. These data were only used in modeling returns. Farmed salmon production for all of Scotland was used in modeling [28], because regional production data were not available.

### Scotland count data.

We also used counts of Atlantic salmon of all ages returning to rivers from 1960–2001 in Scotland from Thorley et al (2005) [29]. The fish counters are maintained by Fisheries Research Services or by Scottish and Southern Energy plc. There were two exposed populations. One is from the Awe Barrage, which empties into a bay with numerous salmon farms. The other is from the Morar River, which is less than 20 km from the nearest salmon farm, in an area of the coast with many farms [8]. Salmon from the control rivers (on the east coast) do not pass by salmon farms in Scotland because of the direction of their migration routes [30], unless they approach the Norwegian coast. There were ten control populations from the following rivers: Aigas, Beanna, Torr Achilty, Dundreggan, Invergarry, Logie, Westwater, Cluni, Erich, and Pitlo. Farmed salmon production for all of Scotland was used in modeling [28] because regional production data were not available.

### Ireland Atlantic salmon.

Estimates of marine survival to one sea winter for hatchery (and two wild) Atlantic salmon populations from Ireland and Northern Ireland (1980–2004) were collected and reported by the ICES Working Group on North Atlantic Salmon [28]. Because only survival estimates are provided, these data were only used in the survival analysis. Salmon from hatcheries on the Screebe, Burrishoole, Delphi, and Bunowen Rivers were considered exposed. Populations from hatcheries on the Shannon, Erne, Lee, Bush, and Corrib Rivers, plus wild populations from the Bush and Corrib Rivers were used as controls.

Production data were not available on a regional basis, so national values [28] were apportioned to bays into which exposed rivers empty by assuming that 30% of national production is in the Kilkieren Bay, 10% is in Clew Bay, 5% is in each of Killary Harbour and Ballinakill Bay. These proportions are based on maps of salmon farm locations from the Irish Marine Institute [25], and they approximately match stock numbers collected by the Central Fisheries Board in the years for which stock numbers are available (P. Gargan, personal communication). Years in which each bay was fallowed were obtained from the Central Fisheries Board (P. Gargan, personal communication), and in these years, the fallowed bays are assigned a production of zero. All exposed rivers empty into bays with salmon farms [25], while control rivers are at least 55 km away from the nearest farm.

### Newfoundland, Canada.

Two data sets from Newfoundland were examined—marine survival estimates of wild Atlantic salmon from four rivers from 1987 to 2004 were used in the survival analysis, and grilse returns to 21 rivers from 1986 to 2004 were used in the returns modeling [31]. Salmon farming in Newfoundland is confined to Bay d'Espoir on the south coast [32] (http://www.fishaq.gov.nl.ca/aquaculture/pdf/aqua_sites.pdf). Only the Conne River (in Bay d'Espoir) was considered exposed; the Little River (also in Bay d'Espoir) was excluded because it has been regularly stocked [31]. The Exploits and Rocky Rivers were also removed from the analysis because of stocking [33]. This left three control rivers for the survival analysis: the Campbellton River, the Northeast Brook (Trepassey), and Western Arm Brook. For the returns analysis, there were 18 control rivers: Campbellton, Crabbes, Fischells, Flat Bay Brook, Highlands, Humber, Lomond, Middle Brook, Middle Barachois, Northeast Brook (Trepassey), Northeast (Placentia), Northwest, Pinchgut Brook, Robinsons, Salmon, Terra Nova (upper and lower), Torrent, and Western Arm Brook. Salmon from control rivers are very unlikely to pass salmon farms because of the direction of their migrations [34]. Farmed salmon production data are from Fisheries and Oceans Canada (DFO) Statistical Services [32].

### New Brunswick and Nova Scotia, Canada.

We compared Atlantic salmon returns to six rivers in the Bay of Fundy (New Brunswick and Nova Scotia, Canada) to returns to four rivers from other areas of New Brunswick and Nova Scotia. We grouped the six exposed rivers into three groups and estimated the impact of aquaculture on each group separately, because salmon from these three groups have different degrees of exposure to salmon farming. The three groups of exposed rivers are the inner Bay of Fundy group (Stewiacke and Big Salmon Rivers), the Saint John River group (Saint John and Nashwaak Rivers), and the outer Bay of Fundy group (St. Croix and Magaguadavic Rivers). Salmon farming in New Brunswick is highly concentrated in the Quoddy region of the outer Bay of Fundy (http://www.gnb.ca/0177/10/Fundy.pdf), although some farms are also found along the Nova Scotia coast of the Bay of Fundy. Salmon from control rivers enter into the Atlantic directly (LaHave River) or into the Gulf of St. Lawrence (Restigouche River, Miramichi River, Catamaran Brook) and do not pass by farms during their migrations. The same controls are used for all comparisons in New Brunswick and Nova Scotia. The estimates of returns to the rivers are published by DFO [28,35–40]. Outer Bay of Fundy salmon must pass through an area containing many salmon farms early during their migrations [41]. Although Saint John River salmon enter the ocean in an area without salmon farms, they are known to pass through the region containing many farms early during their migrations [41]. Salmon from inner Bay of Fundy rivers are considered exposed to salmon farming despite being up to 260 km away because of historical information indicating that juvenile salmon from these populations are found during the summer and fall in the area where salmon farms are currently located [42]. However, the evidence that this region is important habitat for inner Bay of Fundy and Saint John River populations is mixed [43]. For this reason, we ran an alternative model with only outer Bay of Fundy populations considered exposed, and all other New Brunswick and Nova Scotia rivers as controls.

For all New Brunswick rivers, an estimate of egg deposition was used as an index of spawners, to account for a significant increase in the age of spawners in many rivers over the study period. The number of grilse (salmon maturing after one winter at sea) and large spawners (repeat spawners or salmon maturing after two or three winters at sea) in each year was multiplied by a river-specific estimate of fecundity for a salmon of that size. Then, the index of spawners in a given year was derived by adding up all the eggs that could produce smolts in a year *y*, using river-specific ages at smolting from the literature. Returning hatchery-origin spawners are also added to the “spawners” but not to “returns.” “Recruits” is the number of grilse that return to each river in year *y* + 1, so that 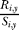 (in Equation 1) is the number of grilse returning per egg that would have smolted in year *y*. Estimates of returns to rivers from traps and other surveys were used in the returns analysis. No corrections were made to account for marine fisheries, but marine exploitation has been quite limited since the late 1980s, when salmon farming became a substantial industry [44]. Farmed salmon production data are from DFO Statistical Services [32].

### British Columbia, Canada, coho salmon.

For coho salmon in British Columbia (BC), spawner estimates are based on DFO's escapement database (NuSEDS), which includes estimates of spawning salmon of all species for hundreds of rivers and streams on the BC coast since 1950 (P. VanWill, DFO Pacific, unpublished data). We considered rivers on the east side of the Queen Charlotte and Johnstone Straits to be exposed (all rivers from Wakeman Sound to Bute Inlet, DFO Statistical Areas [SAs] 12 and 13). All rivers on the BC Central Coast from Finlayson Channel to Smith Inlet (SAs 7, 8, 9, and 10) were included as controls. In the regions considered exposed in BC, all salmon must pass by farms to get into the open ocean, although in some cases, the farms are at the end of long channels down which the salmon migrate (as far as 90 km in the most extreme case). Control populations to the north do not pass by farms, because of the direction of their migration routes [45].

Coverage in the NuSEDS database varies considerably in time and space, as does the quality of the estimates. We changed all indicators of unknown values (including “none observed” and “adults present”) to a common missing value indicator. To reduce effects of inconsistent monitoring procedures, only data since 1970 were included in the analysis. All rivers known to be regularly stocked with hatchery salmon or to contain constructed spawning channels were also removed from exposed and control areas, leaving 49 exposed and 70 control rivers. Estimates were combined for each SA, the smallest areas for which catch rates are estimated. This was done by modeling returns to each SA and year, using a generalized linear model with negative binomial errors. The predicted returns for each SA were then used as spawner estimates (*S_i_*_,*y*_ in Equation 1). To derive recruitment estimates, we followed Simpson et al. (2004) [46], applying exploitation rate estimates from Toboggan Creek (J. Sawada, DFO Pacific, personal communication) to the controls, and the average of the exploitation rates for Quinsam Hatchery, Big Qualicum Hatchery, and the Black Creek wild indicator population to the exposed stocks. After 1998, only the estimates from Black Creek were used for exposed stocks. Recruitment estimates for coho were based on the assumption that coho follow a fixed 3-y life cycle.

For pink, chum, and coho salmon, aquaculture production estimates include all salmon species farmed in SAs 12 and 13 (the Queen Charlotte and Johnstone Straits) from 1990 to 2003 (H. Russell, BC Ministry of Agriculture, Food, and Fisheries, unpublished data). In years when two or fewer companies were raising salmon in either area, estimates were not available. BC salmon farm locations are made available at http://www.al.gov.bc.ca/fisheries/licences/MFF_Sites_Current.htm.

### British Columbia, Canada, pink salmon.

Estimates of pink salmon spawner abundance were derived in the same manner as described above for coho salmon. “Returns” are spawners plus catch for a given year, assuming a fixed two year life cycle. The same regions were considered exposed, but because enumeration varies by species, there were only 36 exposed rivers from SAs 12 and 13 (from Wakeman Sound to Bute Inlet) included. Wood et al. (1999) [47] consider the pink salmon catches in SAs 8, 9, and 10 to consist mainly of salmon returning to those areas (respectively), so catch data from DFO [48] were used in each of these SAs. Area 7 was excluded from the survival analysis because catches for SA 7 are difficult to estimate due to the adjacent regions being much larger [47], leaving 47 control rivers from Burke Channel to Smith Inlet.

For Queen Charlotte and Johnstone Straits (the exposed areas), DFO does not estimate catches at the level of individual SA. To obtain approximate returns to each exposed SA, we found the proportion of total escapement to the Straits that was in our dataset (i.e., regularly enumerated rivers on the east side of the Straits without a major hatchery or constructed spawning channel) and assumed the same proportion of the total catch would be returning to those rivers (i.e., assumed equal catchability across stocks). For odd years, we used estimates from the Pacific Salmon Commission (B. White, unpublished data) of the catch of pink salmon in Johnstone and Georgia Straits that were not returning to the Fraser River. In even years, there is no pink salmon run on the Fraser River, so total returns to the Straits could be used.

### British Columbia, Canada, chum salmon.

For chum salmon, we used estimates of returns (i.e., before exploitation) and spawners to large coastal areas [49]. Chum from the east side of Queen Charlotte and Johnstone Straits, from Wakeman Sound to Bute Inlet (SAs 12 and 13) were considered exposed to salmon farming, while chum from the Central Coast from Bute Channel to Seymour Inlet (SAs 8–11) were considered controls. Estimates were available as a single time series for the exposed area, and a time series for each SA for the controls. An index of recruits per spawner was generated by lining up returns with spawners according to age distributions given in Ryall et al. (1999) [50], to 1998, and then the average values from 1988–1998 for the subsequent years, to 2003.

## Supporting Information

Figure S1Survivals of Salmonids in Control (Black) and Exposed (Blue) Stocks, along with Aquaculture Production (Red)The returns have been summarized by a multiplicative model 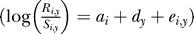 ; the mean survival across stocks for each year is plotted. Survivals for exposed Saint John River stocks have been multiplied by 10 for clarity (dashed line). Survival is estimated across different portions of the life cycle in different regions; from smolt to adult for Irish salmon and Newfoundland, from egg to adult for Bay of Fundy and Saint John River stocks, and from adult to adult in BC stocks.
(15 KB PDF)

Table S1 Results of Alternative Models for the Survival Analysis Effect size estimates (*y*'s) and their standard errors have been multiplied by 10^3^, 10^4^, or 10^8^ (as labeled), to make numbers easier to read.
(22 KB PDF)

Table S2 Results of Alternative Models for the Returns Analysis Effect size estimates (*y*'s) and their standard errors have been multiplied by 10^3^, 10^4^, or 10^8^ (as labeled), to make numbers easier to read.
(23 KB PDF)

Text S1 Alternative Model Formulations, Including the Bayesian Analysis (58 KB PDF)

## Abbreviations

AIC: Akaike information criteria
BC: British Columbia
DFO: Fisheries and Oceans Canada
SA: statistical area
