# Supplementary material for: A Global Assessment of Salmon Aquaculture Impacts on Wild Salmonids
Source: PLoS Biol. 2008 Feb 12;6(2):e33. doi: 10.1371/journal.pbio.0060033 (PMC2235905; doi:10.1371/journal.pbio.0060033)
Supplement: Supplementary file 1 [file pbio.0060033.sd001.pdf]

Supplementary Methods for *A Global Assessment of Salmon Aquaculture Impacts on Wild Salmonids*

Jennifer S. Ford and Ransom A. Myers

Alternative model formulations

We repeated all analysis in R (MASS and lme packages) and SAS (Proc Mixed, Proc Genmod and Proc GLIMMIX). In all cases the difference in the results were very minor. We also considered a large number of alternative statistical models, six of which are described in Tables S1 and S2.

If we did not include autocorrelated error, the parameter estimates did not change substantially, but the standard errors were smaller because we effectively over-estimated the degrees of freedom. The AIC was generally higher for these models. In general, $\lambda = 0.5$ provided a better fit than $\lambda = 1$ or 2 (Tables S1 and S2).

We also ran a fixed effects model with normal errors after log transformation and with gamma errors with a log link. These models are not directly comparable using the AIC, but always gave similar parameter estimates.

Bayesian Meta-Analysis

Let τ_k^2 be the true, unobserved estimation variance of γ_k. The relationship between the true variance, τ_k^2, and the estimated, s_k^2, is given by

$$\frac{\nu_k s_k^2}{\tau_k^2} \sim \chi^2_{\nu_k}, \tag{1}$$

where ν_k is the degrees of freedom [1]. Thus, our prior for τ_k^2 is given by

$$\tau_k^2 \sim \frac{\nu_k s_k^2}{\chi^2_{\nu_k}}. \tag{2}$$

We used the number of years of overlapping time series as an estimate of the degrees of freedom. To ensure that we were not overestimating the certainty of our estimated variance, we also estimated the model assuming half that number.

We assumed weakly informative priors for α_0 and α_1, i.e. normal distributions with zero mean and relatively large standard deviations. As suggested by

Gelman (2004) [2] we used a uniform prior on the standard deviation of the among study (region) variation.

The Bayesian analysis was carried out using Markov chain Monte Carlo in WinBUGS. One hundred thousand iterations were dismissed as burn-in and the following two hundred thousand iterations were used for parameter estimation. Convergence was assessed using the diagnostics in WinBUGS.

The Bayesian analysis gave 95% credible limits slightly larger than those estimated by maximum likelihood, but in no case were these increases large enough to affect the interpretation of the results.

References

[1] DuMouchel W (1990) Bayesian meta-analysis. In: Berry DA, editor, Statistical Methodology in the Pharmaceutical Industry, New York: Dekker. pp. 509–529.

[2] Gelman A, Carlin JB, Stern HS, Rubin DB (2004) Bayesian Data Analysis. Boca Raton: Chapman & Hall /CRC.
