# Supplementary material for: A Global Assessment of Salmon Aquaculture Impacts on Wild Salmonids
Source: PLoS Biol. 2008 Feb 12;6(2):e33. doi: 10.1371/journal.pbio.0060033 (PMC2235905; doi:10.1371/journal.pbio.0060033)
Supplement: Supplementary file 2 [file pbio.0060033.sg001.pdf]

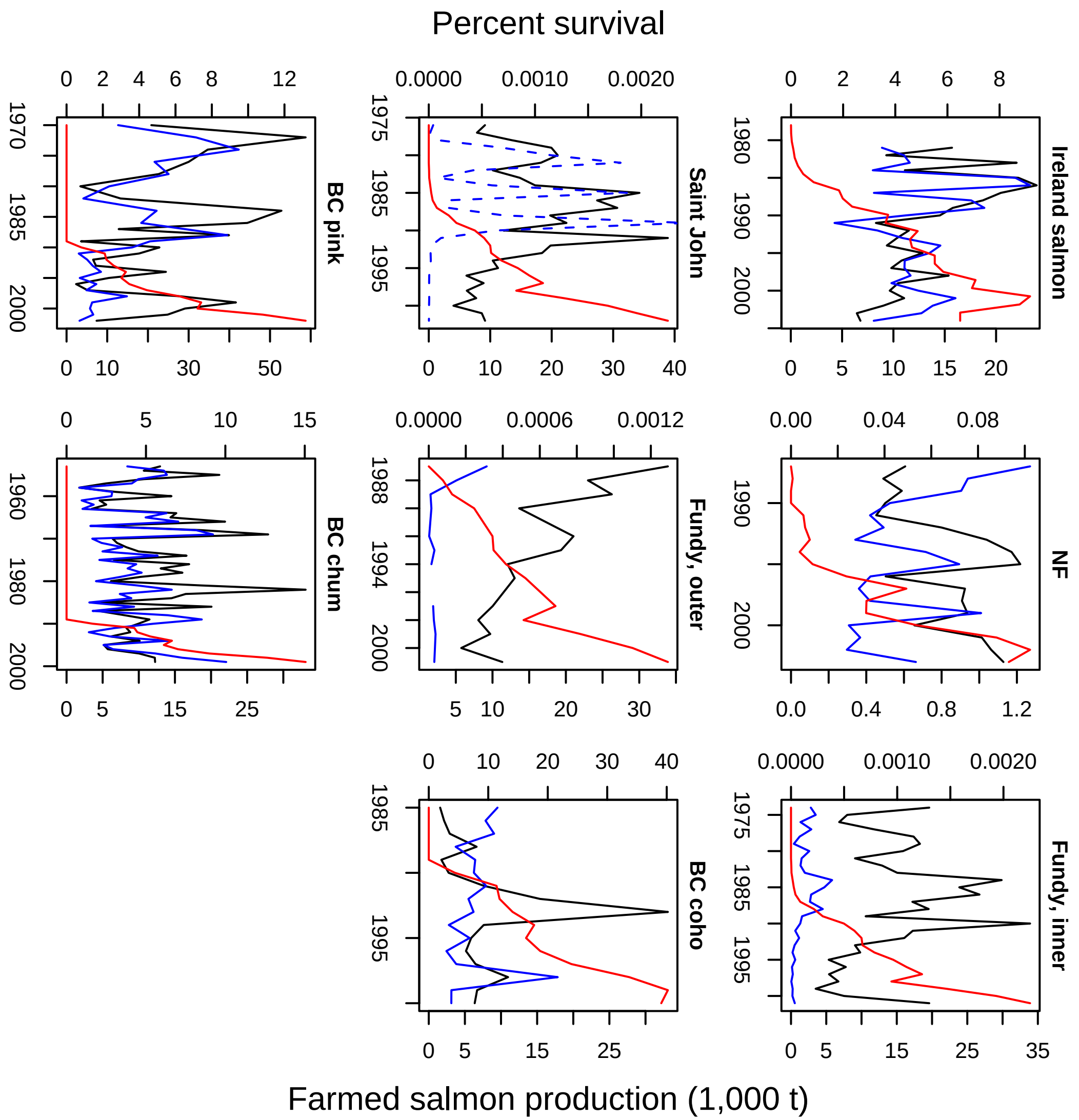
Percent survival
BC pink
Saint John
Ireland salmon
BC chum
Fundy, outer
NF
BC coho
Fundy, inner
Farmed salmon production (1,000 t)
