# Supplementary material for: A Global Assessment of Salmon Aquaculture Impacts on Wild Salmonids
Source: PLoS Biol. 2008 Feb 12;6(2):e33. doi: 10.1371/journal.pbio.0060033 (PMC2235905; doi:10.1371/journal.pbio.0060033)
Supplement: Supplementary file 3 [file pbio.0060033.st001.pdf]

Region		base model $\lambda = 0.5$ x10^3	no autocorrelation x10^3	$\lambda = 1$ x10^4	$\lambda = 2$ x10^8	fixed effects x10^3	all fixed, Gamma errors x10^3
Ireland salmon	γ	-0.95	3.985	0.001	6.242	7.185	4.954
	s.e.	4.633	2.093	7.146	12.382	2.492	1.971
	AIC	593.651	599.885	593.692	596.4	NA	NA
Newfoundland	γ	-27.747	-28.129	-65.724	-430.554	-34.176	-32.489
	s.e.	10.024	7.75	29.541	240.473	8.369	7.917
	AIC	66.905	73.137	68.886	79.508	NA	NA
Fundy, inner	γ	-18.96	-18.678	-11.324	-1.582	-11.461	-12.705
	s.e.	2.412	1.844	1.678	0.76	2.484	2.202
	AIC	316.332	326.726	325.807	343.018	NA	NA
Saint John	γ	-10.498	-10.347	-5.51	-1.012	-6.679	-6.058
	s.e.	2.108	1.155	1.227	0.358	1.853	1.81
	AIC	165.354	177.11	167.068	173.431	NA	NA
Fundy, outer	γ	-25.07	-15.673	-10.2	-2.71	-18.01	-13.469
	s.e.	3.703	2.174	1.123	0.444	3.372	4.356
	AIC	1674.04	161.582	167.682	128.536	NA	NA
BC coho	γ	-1.8	-3.259	-1.348	-0.464	-11.591	-10.911
	s.e.	3.291	2.786	2.102	0.69	4.326	3.422
	AIC	288.503	295.558	288.38	288.502	NA	NA
BC pink	γ	-6.283	-6.183	-3.036	-0.504	-5.305	-4.335
	s.e.	1.749	1.668	0.941	0.205	2.419	2.123
	AIC	368.476	366.908	370.804	375.277	NA	NA
BC chum	γ	-0.538	-0.62	0.553	0.575	-3.311	-3.597
	s.e.	2.177	1.393	1.427	0.484	1.84	1.786
	AIC	246.697	276.783	246.604	248.08	NA	NA
