# Supplementary material for: A Global Assessment of Salmon Aquaculture Impacts on Wild Salmonids
Source: PLoS Biol. 2008 Feb 12;6(2):e33. doi: 10.1371/journal.pbio.0060033 (PMC2235905; doi:10.1371/journal.pbio.0060033)
Supplement: Supplementary file 4 [file pbio.0060033.st002.pdf]

Region		base model $\lambda = 0.5$ x10^3	no autocorrelation x10^3	$\lambda = 1$ x10^4	$\lambda = 2$ x10^8	fixed effects x10^3	all fixed, Gamma errors x10^3
Ireland trout	γ	-18.726	-18.957	-6.797	-1.465	-21.543	-19.825
	s.e.	2.647	2.261	2.187	0.981	2.447	1.783
	AIC	2178.873	2335.682	2255.875	2262.657	NA	NA
Scotland catch	γ	-5.643	-0.309	-1.279	-0.053	-0.094	-0.095
	s.e.	1.394	0.257	0.29	0.013	0.257	0.256
	AIC	-11.15	81.524	-14.483	-12.084	NA	NA
Scotland count	γ	-2.583	-2.621	-0.361	-0.024	-2.067	-2.491
	s.e.	0.97	0.505	0.673	0.044	0.543	0.508
	AIC	569.673	739.618	610.78	610.753	NA	NA
Newfoundland	γ	-16.338	-24.093	24.052	55.454	-25.972	-27.75
	s.e.	7.5	6.214	20.303	48.855	6.243	6.371
	AIC	412.694	498.006	465.314	465.429	NA	NA
Fundy, inner	γ	-23.683	-24.004	-9.467	-1.519	-25.76	-21.586
	s.e.	5.997	2.275	3.281	0.762	2.624	1.575
	AIC	407.231	561.951	410.047	414.056	NA	NA
Saint John	γ	-14.725	-9.891	-4.337	-0.208	-11.302	-7.916
	s.e.	4.121	2.141	2.311	0.52	2.294	1.402
	AIC	296.148	484.079	305.046	308.406	NA	NA
Fundy, outer	γ	-18.69	-53.297	-36.8	-12.221	-14.54	-15.821
	s.e.	1.972	3.372	4.926	14.646	2.271	1.511
	AIC	NA	558.996	738.046	1273.499	NA	NA
BC coho	γ	-0.898	-8.124	-0.094	0.012	-13.84	-11.764
	s.e.	4.081	2.601	1.77	0.257	2.917	2.319
	AIC	269.24	320.049	269.285	269.285	NA	NA
BC pink	γ	-8.426	-7.998	-4.161	-0.751	-7.141	-6.373
	s.e.	2.133	1.89	1.001	0.196	2.012	1.626
	AIC	398.195	404.162	402.525	405.281	NA	NA
BC chum	γ	-3.227	-2.963	-1.299	-0.148	-2.428	-2.627
	s.e.	1.809	1.301	0.9	0.177	1.333	1.261
	AIC	351.759	368.446	355.53	356.854	NA	NA
